# Quilting Sutures in Breast Reduction: A Randomized Controlled Trial of Their Impact on Postoperative Drain Output

**DOI:** 10.1093/asjof/ojaf119

**Published:** 2025-10-23

**Authors:** Alp Ercan, Ersin Yavuz, Serkan Melenkiş, Anıl Demiröz

## Abstract

**Background:**

Quilting sutures (QSs) are used to reduce dead space and may decrease the need for surgical drains. Their application in breast reduction surgery could improve postoperative outcomes and facilitate earlier discharge.

**Objectives:**

The aim of the authors of this study is to evaluate whether QSs significantly reduce postoperative drainage and hospital stay in breast reduction surgery, compared with standard closure without quilting.

**Methods:**

A prospective, randomized, controlled study was conducted involving 87 female patients who underwent bilateral breast reduction between June 2023 and December 2024. Patients were randomized into 2 groups: the QS group (*n* = 38) and control group (*n* = 49). All patients received closed suction drains. Patients were included in the study if complete inpatient follow-up data were available for at least 24 h postoperatively. Primary outcomes included drainage volume on Postoperative day 1 and duration of hospital stay. Secondary outcomes included postoperative complications. Data were analyzed using SPSS v22.0; *P* < .05 was considered significant.

**Results:**

Mean drainage volume on Postoperative day 1 was significantly lower in the QS group (17.6 cc) compared with the control group (36.1 cc; *P* = .017). Hospital stay was also significantly shorter in the QS group (mean 1.02 days) vs controls (1.32 days, *P* = .032). No significant differences were found in infection rates or wound-healing complications between groups.

**Conclusions:**

This randomized controlled trial demonstrated that the use of QSs in breast reduction surgery can significantly reduce postoperative drain output and hospital stay, without increasing complication rates.

**Level of Evidence:**

2 (Therapeutic) 
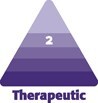

Quilting sutures (QSs; also known as progressive tension sutures when used in a specific setting to alleviate tension) are used widely to be the solution to problems stemming from the presence of dead spaces.^1^ They have become popular in various cosmetic procedures ranging from body shaping surgeries to facelifts.^2,3^ Because they are efficient means to obliterate dead spaces, QSs can also be a factor in avoiding surgical drains. The use of surgical drains has traditionally been common practice in breast surgeries; however, recent systematic reviews and professional guidelines, including those from the American Society of Plastic Surgeons, suggest that drains do not significantly reduce complications such as seroma or hematoma, and a growing number of surgeons are adopting drainless techniques.^4-6^ Dušková et al in a 2024 systematic review analyzing 13 studies with over 1100 breasts concluded that complication rates were comparable between drained and undrained groups, whereas the undrained group benefited from shorter hospitalization and improved comfort.^7^ In addition to that, they can be a nuisance for both the patient and the provider. Primary patient complaints about using surgical drains are pain and associated discomfort, but more importantly, they can be a factor in restricting ambulation and can even delay discharge from the facility.^8^ Based on main author's personal communications with his patients and various colleagues throughout his career, the surgical drains were pointed out as significant deterrents to early patient discharge following breast reduction.

As most surgical procedures, aesthetic surgery is coursing for a more cost-efficient way with the help of improved perioperative anesthesia management, postoperative recovery protocols, and overall improved safety.^9^ Breast reduction is one of the most common aesthetic surgeries overall, and multiple studies are being published proposing performing breast reduction as an outpatient surgery rather than 1 necessitating an overnight stay.^10-12^ A large cohort study involving 18,780 patients confirmed that breast reductions can be safely performed on an outpatient basis without increased risk.^13^ Previous studies have shown that when QSs are used during abdominal closure, drains can be omitted, with improved outcomes in both abdominoplasties and deep inferior epigastric perforator (DIEP) flap reconstructions.^14,15^ The primary author routinely uses QSs in breast reductions with a high amount of tissue resections and experienced significantly decreased discharge. Furthermore, many patients have experienced close-to-zero discharge in their drains at Postoperative day 1. We wanted to investigate whether this phenomenon is a reliable observation or a subjective deduction and designed a prospective study for quantifiable data. To our knowledge, there has not been any previous prospective study in breast reduction evaluating the effect of using QSs on the amount of fluid discharge from the operation site after the surgery. We further aim to evaluate the extended effect of QSs on patient discharge patterns.

## METHODS

### Design

A prospective, randomized, controlled factorial study was designed to evaluate the fluid collection reducing capabilities of QSs in breast reduction and compare them with standard protocol breast reduction with the absence QSs. As secondary objectives, the amount of hospital stay and complications in the early postoperative period were also investigated. Two study groups were planned: Group C (control group; drains are placed and no QSs were used), Group QS (drains are placed and QSs are used). The allocation for treatment was randomized.

### Patients and Settings

After obtaining ethical approval from the Istanbul University-Cerrahpaşa Medical Research Ethics Committee (approval number: W3z4L9pz), consecutive nonsmoking female patients over the age of 16 with a BMI below 30, presenting with breast hypertrophy and meeting the inclusion/exclusion criteria, were selected for the study.” Patients were included in the study if complete inpatient follow-up data were available for at least 24 h postoperatively. Exclusion criteria included: morbid obesity (BMI ≥30), active smoking defined as consumption of more than half a pack of tobacco daily, uncontrolled diabetes mellitus, active soft tissue diseases (eg, rheumatoid arthritis and systemic lupus erythematosus), and the presence of >1 risk factor for wound complications. Additionally, patients without complete postoperative follow-up data for at least 24 h were excluded. All patients underwent surgery by the same team between June of 2023 and December of 2024. Randomization was performed using a computer-generated simple random number sequence with a 1:1 allocation ratio. No blocking or stratification was applied. Allocation was concealed using sealed opaque envelopes that were opened at the time of surgery. Allocation concealment was ensured using sequentially numbered, opaque, sealed envelopes prepared by an independent staff member not involved in the surgical procedures. The envelopes were opened in the operating room after patient preparation, just before the closure phase began. Because of the nature of the intervention, surgeons were not blinded. However, patients were not informed of their group allocation, and drain output measurements and complication assessments were performed by ward staff not involved in the surgical procedures and unaware of treatment allocation.

**Figure 1.**
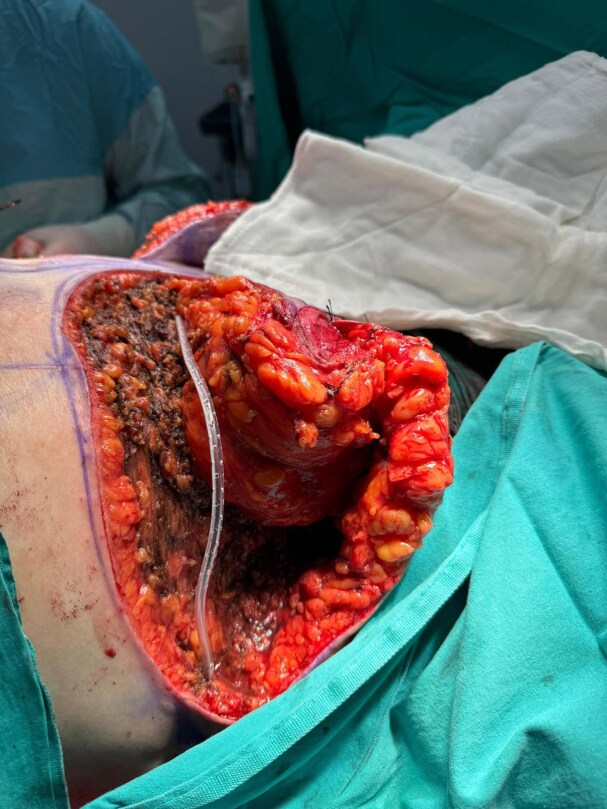
The appearance of the breast pedicle after repositioning in a 34-year-old female patient.

**Figure 2.**
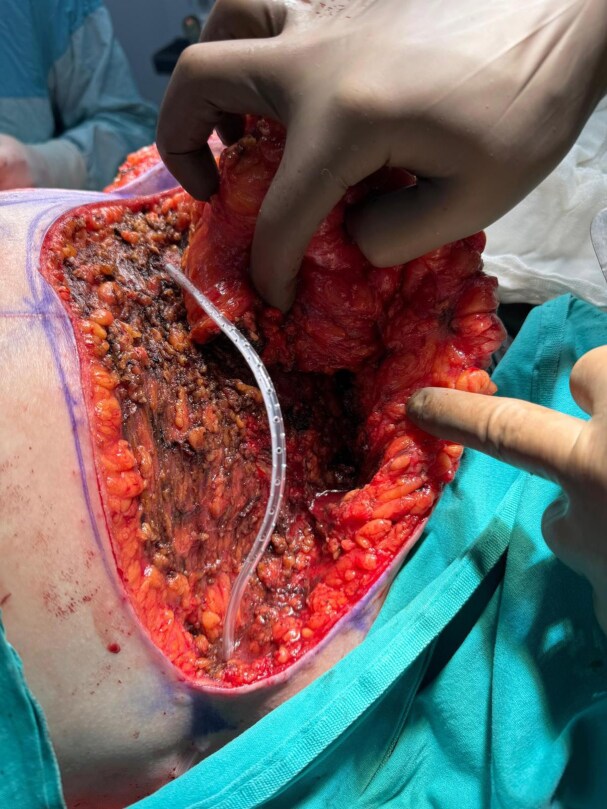
The view of the potential dead space following pedicle placement in a 34-year-old female patient.

**Figure 3.**
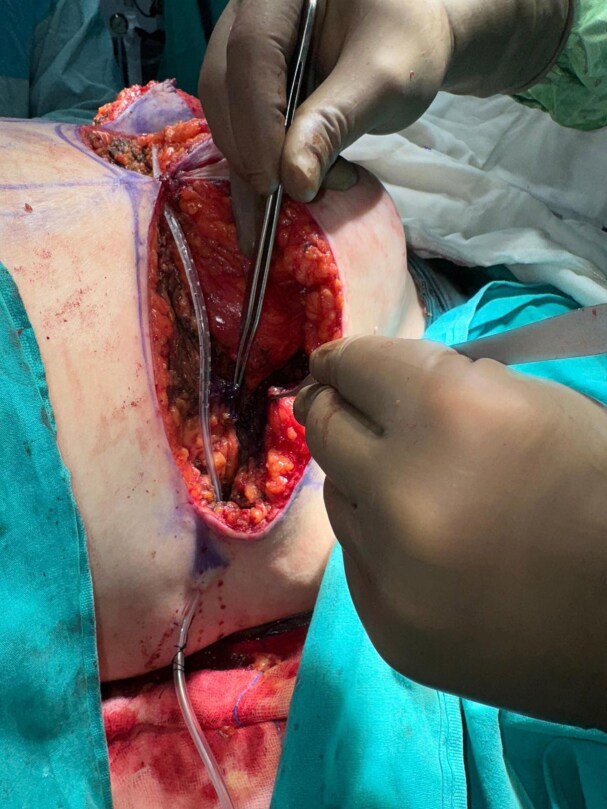
The view of tissues to be approximated using quilting sutures to eliminate the potential dead space in a 34-year-old female patient.

**Figure 4.**
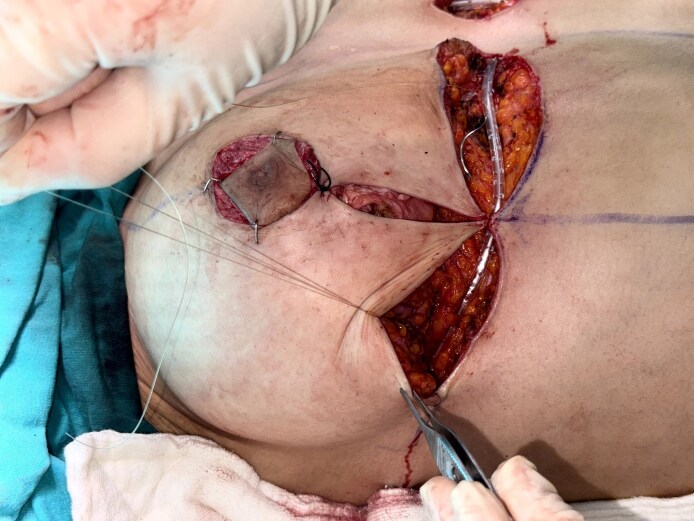
Intraoperative view of the quilting suture application stage in a 34-year-old female patient.

**Figure 5.**
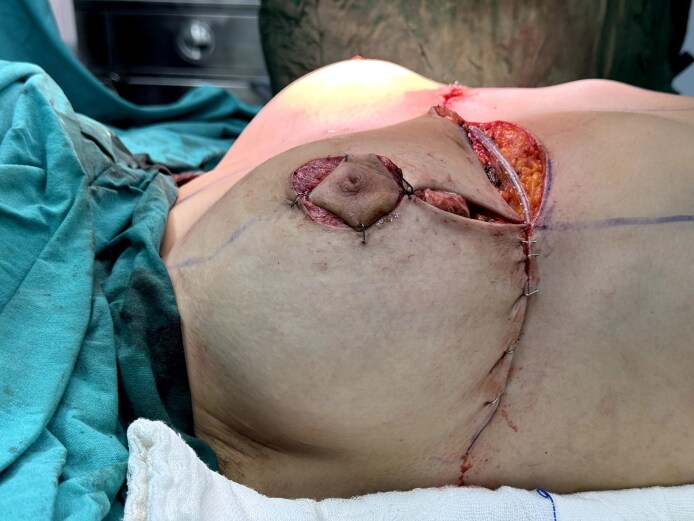
Appearance of the breast following the application of quilting sutures in a 34-year-old female patient.

**Figure 6.**
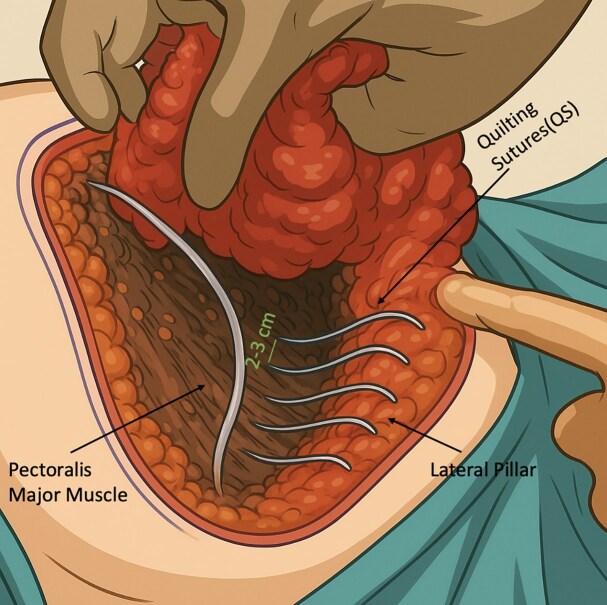
Schematic illustration showing the placement of quilting sutures (QSs) between the lateral pillar and the pectoralis major muscle at 2 to 3 cm intervals during breast reduction surgery. During the preparation of this figure, the authors used ChatGPT-5 (OpenAI, San Francisco, CA) to assist with shading and coloring. After using this tool, the authors reviewed and edited the content as needed and take full responsibility for the content of the publication.

All patients underwent either superior pedicle or superomedial pedicle breast reduction with inverse-T-scar design under general anesthesia with a 1-dose preoperative antibiotic prophylaxis. After skin incisions were made, elevation of the flaps and resection of the excess tissue were performed with electrocautery. After bringing the pillars together, if QSs to be used, 6 to 10 separate fixation sutures were placed between the flaps and the pectoralis major muscle fascia using long-lasting absorbable stiches (monofilament poliglecaprone, 2-0 caliber; Figures 1–5). The sutures were spaced ∼2 to 3 cm apart and primarily targeted the central and inferior pole of the breast tissue, where dead space was most prominent following pillar re-approximation (Figure 6). A total of 6 to 10 sutures were placed per breast, depending on the breast size and contour. This approach ensured adequate obliteration of potential spaces and uniform distribution of tension across the closure site. Throughout all the surgeries, a single closed suction Hemovac drain (14 French tube connecting to a 400 cc collection reservoir) was placed on each side, extending to the axilla and inferior half of the breast and exiting under the axilla. No liposuction was performed for further re-shaping the breast in this study cohort.

### Outcomes and Follow-Up

The amount of fluid accumulated in the drain reservoir was measured daily starting from Postoperative day 1 and onwards. Thirty cubic centimeters and under were accepted as an indication for drain removal. All patients were kept in-service for the night of the surgery. Time to discharge was calculated from the time of transfer to the postanesthesia care unit to the time the patient physically left the hospital facility, as recorded in the hospital electronic system. Patients were discharged after removal of the drains on both breast and then called in bi-weekly for postoperative control and follow-up. Postoperative drainage outputs, duration of drain use, duration of hospital stay, and complications including hematoma were recorded and subsequently compared between Group QS and Group C. To ensure standardized measurement, all drains were emptied at the end of surgery before the patient was transferred to the ward. Drain outputs were measured the following morning at a fixed time of 08:00 Am. The fluid collected in the 400 cc reservoirs was carefully aspirated using 60 cc syringes to allow for precise volumetric assessment.

All data of this study were stored locally, and analysis was performed using SPSS version 22.0 (IBM Corp., Armonk, NY). An alpha value of .05 was used as the cutoff for tests of statistical significance. Outcomes were analyzed using χ^2^ testing methodology with Fisher’s exact test when indicated, with generalized linear models used to assess treatment group difference in, amount of drainage on the first postoperative day, the amount of time for a presence of at least 1 drain, amount of hospitalization days before discharge, incidence of hematoma, wound infection, and wound-healing problems. Patients with who were lost to follow-up were excluded from the final analysis. Given the exploratory nature of this single-center study, no a priori power analysis or sample size calculation was performed. The study was designed to evaluate real-world trends in drainage output and inpatient stay in a prospective manner. Multivariate analysis using generalized linear models was applied to control for potential confounding variables (such as age and resection weight). Although more complex modeling (eg, interaction terms or stratified subgroup analyses) may offer additional insight, they were not implemented because of the limited sample size and the study's exploratory intent. In addition to *P*-values, 95% CIs were calculated and reported to provide estimates of precision and clinical relevance for key outcome measures. For continuous variables such as drain output and hospital stay duration, normality was assessed using the Shapiro–Wilk test. Because data were not normally distributed, comparisons between groups were performed using the Mann–Whitney *U* test. Categorical variables, such as wound infection or dehiscence, were compared using the χ^2^ test or Fisher's exact test where appropriate. Additionally, generalized linear models were employed to control for confounding variables such as age and resection weight.

## RESULTS

**Figure 7.**
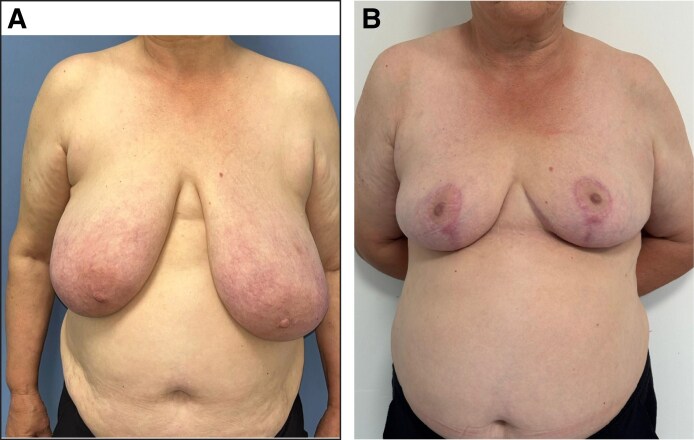
(A) Preoperative and (B) postoperative views of a 44-year-old female patient who underwent bilateral breast reduction with quilting sutures. Postoperative image was taken at 6-week follow-up. Resection weights were 690 g (right) and 720 g (left). Drain outputs on Postoperative day 1 were 16 cc (right) and 18 cc (left). Drains were removed on Postoperative day 1, and the patient was discharged on the same day.

**Figure 8.**
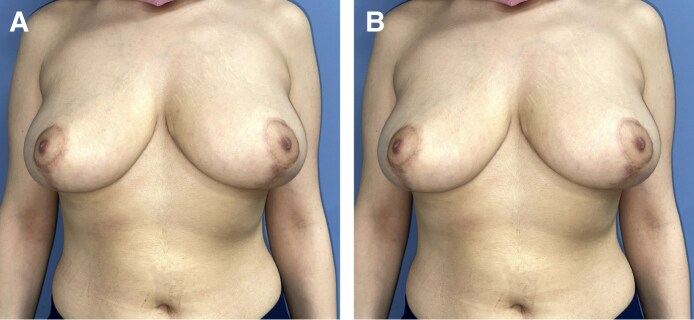
(A) Preoperative and (B) postoperative views of a 34-year-old female patient who underwent bilateral breast reduction with QSs. Postoperative image was taken at 3 month follow-up. Resection weights were 540 g (right) and 580 g (left). Drain outputs were 15 cc (right) and 17 cc (left) on Postoperative day 1. Both drains were removed on Day 1, and the patient was discharged the same day.

Between June 2023 and November 2024, over an 18 months’ period, 100 patients underwent breast reduction surgery. A total of 13 patients were lost to follow-up: 9 from the QS group and 4 from the C group. These patients were excluded from the final analysis, and a per-protocol analysis was performed. As a result, 87 patients were included in the study. Thirty-eight patients had QSs during the surgery and made up the QS group (patient examples from group QS are examined in Figures 7, 8). Forty-nine patients had a standard closure and did not receive QSs and named as the Group C. Patient ages were distributed as similarly between 2 groups without any meaningful statistical difference, with a mean value of 45.8 years. The mean amount of resected weight per breast was 649 g in the study (320-1240 g). There was a slight difference between group QS (mean weight 661 g) and Group C (mean weight 640 g), but the difference was statistically insignificant (*P* = .32). Fifty-two patients had a superior pedicle breast reduction and 35 patients had a superomedial pedicle breast reduction. The mean amount of distance the nipple–areola complex was transferred was 12.7 cm (5-21 cm); groups did not demonstrate any difference in this regard (a complete summary of preoperative and perioperative data is shown in Table 1).

**Table 1.** Demographic Patient Data

Pre- and perioperative patient data	Group QS (*n* = 38)	Group C (*n* = 49)	*P*- value
Age			
Median	39 (IQR: 31-53)	45 (IQR: 34-58)	.08
BMI			
Median	28 (IQR: 26-30)	30 (IQR: 27-32)	.06
Resection weight (per breast)			
Median	661 g (IQR: 490-880)	640 g (IQR: 480-870)	.32
Distance between native and neo-nac position			
Median	14.1 cm (IQR: 10-17)	11.6 cm (IQR: 9-15)	.001*
Comorbidities			
HT	5 (13.2%)	14 (28.6%)	.017*
DM	8 (21.1%)	11 (22.4%)	1
Tobacco usage	4 (10.5%)	7 (14.3%)	.749
Soft tissue disease	1 (2.66%)	3 (6.1%)	.629

The table presents the distribution of demographic characteristics of patients across the study groups. Asterisks indicate statistically significant differences. A *P*-value of <.05 was considered statistically significant. DM, diabetes mellitus; HT, hypertension; Neo-NAC, newly reconstructed nipple–areola complex.

**Figure 9.**
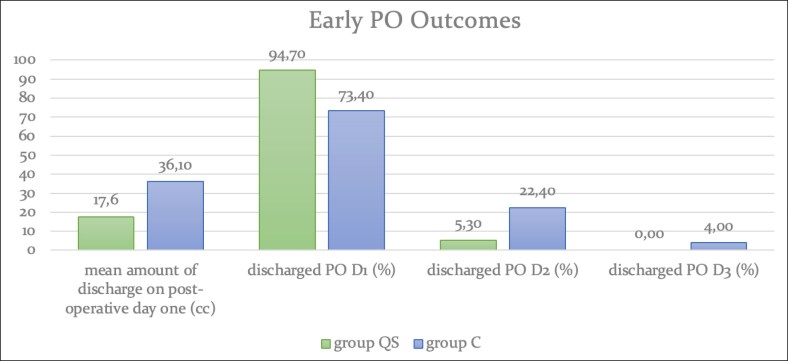
Mean amount of drainage output in on Postoperative day 1 and amount of days patient spent in the facility after surgery.

The mean amount of fluid collection inside the drain bulb at the postoperative first day was 17.6 cc for Group QS and 36.1 cc for Group C (amount of drainage and inpatient stay data is illustrated in Figure 9). In a multivariate model, the presence of QSs was strongly associated with differences in drain output after controlling for resection weight and age (*P* = .017). In 2 patients from Group QS, drains were kept for a total of 2 days and taken out at the Postoperative day 2. On the other hand, for Group C, 11 patients’ drains were kept for 2 days and in 2 patients’ drains were kept for 3 days. As hospital stay was directly related to the presence and duration of drain placement, the significantly reduced drain output observed in the QS group resulted in earlier drain removal and thus shorter hospital stay (*P* = .032). Length of stay was recorded in calendar days (ie, Postoperative day 1, 2, or 3), and not by the hour. Therefore, mean values were rounded to reflect actual discharge timing without implying undue precision. We acknowledge that factors such as administrative processing may cause variations in actual discharge time not reflected in these data. During the follow-up no patients in the study group developed hematomas or major infections necessitating hospitalization and IV therapy. No statistically meaningful difference was found between 2 groups for infection rates and wound-healing problems in a major capacity. Minor wound problems were encountered slightly less in Group QS but the difference was not significant (*P* = .62; statistical analysis of postoperatively collected data is summarized in Table 2).

**Table 2.** Postoperative Outcomes

Postoperative outcomes	Group QS	Group C	*P* value	95% CI of difference
Amount of drainage on Postoperative day 1 (cc)	17.6	36.1	.017*	6.4-33.7
Mean inpatient stay (days)	1.02	1.2	.032*	0.03-0.57
Minor postop infections treated in outpatient setting with oral ab	3	2	.7	RR: 1.93 (0.34-10.79)
Wound dehiscence treated without surgical intervention	5	4	.4	RR: 1.61 (0.44-5.86)

This table shows the statistical comparison of postoperative drainage, complication rates, and length of hospital stay between the groups. Asterisks indicate statistically significant differences. A *P*-value of <.05 was considered statistically significant. AB, antibiotic; QS, quilting suture; RR, risk ratio.

## DISCUSSION

Drainless procedures are becoming more popular and are presented as standard of care even in complicated surgeries such a DIEP flaps.^16^ Perception of having drains after surgery is found out to be bothersome by both the patients and the providers in a recent study.^17^ Patients routinely expressed concerns about drain-site pain, discomfort, and tugging on clothing and both the providers and patients believed that drains contribute to surgical-site infections. Drains are effective tools in following up the accumulation of blood and fluid collection in the postoperative period. They are also helpful in bringing together surgically separated and manipulated layers by maintaining constant negative pressure. Despite the benefits, they can also make ambulation quite distressing for the patient over the fear of ripping off the drains, and they can also be an annoying source of pain.^17^ These factors can ultimately become a concern as we urge these group of patients to be up and walking immediately after the surgery, both for the sake of rapid recovery and prevention of a blood clot.^18^ This discomfort even can become a burden if multiple drains are present in combined procedures such as increasingly popular “mommy make-overs.” Postoperative comfort and regaining the sense of normalcy early as possible is very important for cosmetic patients.

Placing a drain practically may change a day surgery to an overnight stay at the facility. Although by itself the presence of a drain does not necessitate hospitalization but many practitioners tend to keep the patient for following up on the amount of drainage and a majority of patients hesitate to be discharged with their drains intact.^19^ This extra night is a potentially avoidable cost on the health system or on the patient depending on the healthcare system or facility. Counting in the amount of breast reductions performed yearly, it is a substantial sum that can be avoided or at least decreased. Because the study is done in a public health facility in a country where universal health insurance is provided, the cost-effectiveness analysis of drainless breast reduction cannot be investigated objectively, but it may be a potential subject of a future study. The other downsides of placing a drain, albeit quiet minor, are the pain from the removal of the drain and the fear of drain removal.^17^ Overall, the use of QSs consistently and reliably kept the amount of drainage under 30 cc over the course of Postoperative day 1, therefore nullifying the need to place a closed suction drain in the operation site. Although the absolute difference in drain output between groups (∼17 cc) may appear modest, it holds practical clinical relevance in our institutional setting, where discharge is closely linked to achieving drainage volumes under 30 cc. This threshold was consistently reached by the QS group on Postoperative day 1, allowing for significantly earlier discharge. It is important to note, however, that this reduction in hospital stay should be interpreted in the context of our institution's discharge protocol, which requires drains to be removed before discharge. Therefore, the observed shortening of inpatient stay is likely mediated by earlier drain removal resulting from lower output, rather than a direct effect of QSs on discharge timing. Although we acknowledge that many surgeons opt not to use drains in straightforward breast reductions, institutional protocols and local practice patterns vary. In our public teaching hospital, routine drain placement remains standard, and any technique that accelerates safe drain removal translates into meaningful improvements in patient throughput, comfort, and system efficiency. These findings also support the feasibility of a drainless approach in breast reduction procedures when QSs are used effectively to obliterate dead space. Supporting this, a 2025 retrospective study involving 944 female patients found that although drain use slightly reduced seroma rates, it increased infection risk and did not affect length of stay—while over half of the patients had no drains with acceptable outcomes.^20^ Earlier discharge enabled by reduced drain dependency may improve patient comfort and postoperative recovery and may also contribute to cost-efficiency in surgical care depending on institutional reimbursement models.

Future studies with larger cohorts and longer follow-up are warranted to validate these results and assess long-term complications, including seroma formation, fat necrosis, and aesthetic outcomes. If confirmed, QSs could be considered a standard adjunct technique in reduction mammoplasty to optimize perioperative outcomes. Moreover, a recent study demonstrated that, following breast reduction surgery, an increase in BMI was associated with higher seroma rates and a greater need for drain usage, highlighting the importance of techniques that can effectively reduce dead space in high-risk populations.^21^ In addition to significantly expediting patient discharge from the facility, many of the patients with QSs can be sent home the same day if recovery from general anesthesia is adequate. In our study, all the patients were found to be fully awake and alert enough to be discharged on the night of the surgery. It can be argued that discharging the patient with drains can also be an option and we, as authors of the study, routinely do so. Having said that, it may bring forward some time-consuming concerns. On one hand, the patient may be reluctant or unable to empty and reconnect the bulb properly. On the other hand, the patient can be fully capable but taking care of drains in a house setting is a safety concern with multiple unknowns such as animals, kids, house conditions, etc.

The most common deciding factor for using a surgical drain is the presence of a dead space. Therefore, obliteration of dead space may allow us to avoid drains: no dead space, no drains. Despite evidence-based data, surgeons continue to use prophylactic drainage.^22^ Randomized controlled trials have continually failed to show a benefit to support the routine use of closed suction drains in many different plastic surgery procedures. Despite the lack of evidence, drains continue to be used after elective surgeries including breast reduction.^23^ Plastic surgery is not the only specialty guilty of using drains despite evidence contradicting its use. Randomized controlled trials have continually failed to show a benefit to support the routine use of closed suction drains in many different surgical settings.^24,25^ Despite the lack of evidence, drains continue to be used after elective procedures.

Like similar prospective studies, the limitation of the study was its relatively low patient number. It is hard to obtain concrete data about major complications in a prospective study about a low-complication surgery such as breast reduction unless reaching a very large cohort. It is also important to note that no control group without drains was included. We have failed to reliably investigate whether the presence of QSs has any effect on early surgical complications. Our main aim for this study was to investigate whether it is plausible to discard surgical drains while utilizing QSs to obliterate the dead space and we concluded that proper usage of QSs consistently decreases the amount of drainage and eliminates the need for surgical closed suction drains and a drainless approach can be considered. In further studies, we plan to investigate operation times, complication rates, and reoperation as covariates in the meta-regression. Another point to underline can be the effect of quilting on emphasizing the natural lateral curvature of the breast in the immediate postoperative period. We believe its effect on long-term results is negligible or, at the least, cannot be commented unless a long-term study with a higher number of participants can be performed.

Breast reductions can be safely performed as day surgeries, and the presence of a drain may influence discharge decisions. One of the main things considered is whether to place a drain is the presence of an empty space, and if that empty space can be avoided, there would not be a need to place surgical drains. Placement of QSs to obliterate empty space is an effective method, and it can easily be learned and replicated and does not increase the surgical time considerably. This prospective study aims to provide evidence to supporting the obsolescence of drains in breast reductions and furthermore to evaluate the feasibility of omitting the breast drain when QSs are used regularly.

This study has several limitations. First, although our findings support the use of QSs in reducing postoperative drainage, the study did not include a control group without drains. This was because of institutional protocols at the time, which mandated drain placement in all breast reduction procedures, making it ethically unfeasible to omit drains without previous supporting data. Second, the follow-up period was limited to the early postoperative phase (first 3 weeks), which may not be sufficient to capture delayed complications such as fat necrosis or late seroma formation. Third, the loss to follow-up was disproportionately higher in the QS group. Although the study was conducted using inpatient data, it is unclear how some patients were excluded. No predefined minimum follow-up period was described in the Methods section, which we acknowledge as a design limitation.

Fourth, the study did not include a priori sample size calculation. This was a single-institution exploratory study aimed at assessing real-world trends in postoperative drainage and discharge timing. Although statistically significant differences were observed, the study may not have been adequately powered to detect minimal clinically important differences in fluid output or inpatient stay. Future studies with larger sample sizes and formal power analysis will be required to confirm these preliminary findings.

Fifth, this study utilized a per-protocol analysis, excluding 13 patients who were lost to follow-up (9 from the QS group and 4 from the control group). This exclusion may introduce potential bias and affect the generalizability of the results. Future studies should aim for more robust follow-up strategies to minimize attrition bias. Collectively, these factors may limit the generalizability of our findings and underscore the need for further large-scale, long-term, prospective studies to validate these results.

## CONCLUSIONS

This prospective, randomized controlled study demonstrates that the use of QSs in breast reduction surgery significantly reduces postoperative drainage volume without increasing complication rates. In our institutional setting, where discharge timing is directly tied to drain removal, this reduction in drainage was associated with a shorter inpatient stay. It is important to note that this observed effect on hospital stay is likely mediated by earlier drain removal rather than a direct influence of QSs on discharge decisions. These findings support the role of QSs in reducing postoperative drain burden and facilitating earlier discharge in select patient populations under similar clinical protocols.
